# Supplementary material for: Are carbon emissions trading and green financial instruments synergistic? -Comprehensive quantitative research based on content analysis
Source: PLoS One. 2024 Mar 7;19(3):e0298601. doi: 10.1371/journal.pone.0298601 (PMC10919601; doi:10.1371/journal.pone.0298601)
Supplement: S1 Data — (ZIP) [file pone.0298601.s001.zip › Data/地级市Z_F工作报告.zip!/║■▒▒/╧╠─■╩╨/└┤╫╘╣1⁄2╓┌║┼í╛┬φ┐╦╩2╛▌═°í┐_2012.docx]

各位代表：

现在，我代表第三届市人民政府，向大会报告工作，请予审议，并请各位市政协委员和列席人员提出意见。

一、过去四年工作的回顾

2008年以来，在省委、省政府和市委的坚强领导，市人大、市政协和社会各界的监督支持下，市政府团结带领全市人民，高举中国特色社会主义伟大旗帜，深入贯彻落实科学发展观，以开放促发展，以改革求突破，全力推进鄂南经济强市建设，市三届人大历次会议确定的各项任务圆满完成。

（一）经济发展持续攀升。面对复杂的经济形势和罕见的自然灾害冲击，坚持科学发展不动摇、弯道超越不松懈，扩内需，调结构，保增长，市域经济总体水平大幅提升。

经济实力明显增强。预计地区生产总值640亿元，连续跨越300亿元、400亿元、500亿元、600亿元四个台阶，是2007年的1.8倍，年均增长15.8%。预计全社会固定资产投资550亿元，是2007年的3.97倍，年均增长41.2%；社会消费品零售总额239亿元，是2007年的2.26倍，年均增长22.5%；全地域财政收入、地方财政总收入、地方一般预算收入、市本级一般预算收入分别达113.88亿元、51.51亿元、35.56亿元、10.38亿元，是2007年的2.9倍、2.5倍、2.8倍、3.4倍，年均分别增长30.5%、25.7%、29%、35.7%。

发展方式加快转变。坚持“两强促一强”的发展方略，形成市区、县域竞相发展的良好格局。积极融入“两圈一带”，引进圈内城市项目投资176.3亿元，咸安、嘉鱼、赤壁挤进武汉新港建设规划。着力扩大内需，累计完成全社会固定资产投资1488.7亿元，其中工业投资年均增长50.2%。红牛二期、光宝电子建成投产，咸宁核电、蒲圻电厂二期、红牛三期、黄鹤楼酒业、南玻节能玻璃等项目顺利实施，武咸城际铁路、武咸快速通道、咸通高速、咸黄高速、通界高速、咸崇旅游公路等项目进展良好，武广高铁、杭瑞高速、大广高速建成通车，天然气管道全线贯通。城乡市场繁荣活跃，住房、教育、文化、医疗等消费支出逐年增长，汽车、娱乐、休闲、体育、健身等消费热点持续走旺。大力推进节能减排，关停高能耗、高污染企业203家，市县污水处理工程投入运营，垃圾无害化处理工程加快建设，省定节能减排目标任务全面完成；大力推进生态建设，森林植被覆盖率达到54.2%。荣膺中国人居环境范例奖、全国最适宜人居城市、中国魅力城市、中国温泉之城、中国十大最具成长力创新型城市、全国第二批可再生能源建筑应用示范市和全省首批低碳经济试点市。

产业调整取得实效。工业提速提质。新口径规模以上工业企业620家，其中产值亿元企业230家。预计规模以上工业增加值266亿元，年均增长28%；工业经济效益综合指数320.58。市直机电、咸安苎麻纺织、赤壁纺织服装、通城涂附磨具和通山石材列为全省重点成长型产业集群，联乐床垫、川字砖茶、巨宁地板、澳森地板、九宫绿茶、犀利砂带荣获中国驰名商标。农业丰产丰收。粮食生产“八连增”，实现总产23.58亿斤。嘉鱼获评全国水产百强县，通城列为国家农业综合开发县。特色农产品基地达到350万亩，建成畜禽养殖小区260个，省级农业龙头企业达到28家，“三品”标志认证320个。以旅游为主体的服务业提档升级。预计服务业增加值226亿元，是2007年的1.87倍，年均增长16.8%；连续三届国际温泉文化旅游节培育了“香城·泉都”品牌，提升了咸宁旅游的影响力。全年接待游客突破1500万人次，总收入77.6亿元，分别是2007年的3.5倍和5.1倍。跻身全国五个旅游标准化试点城市之列，获评湖北最受欢迎的国内旅游目的地。房地产投资达到69.34亿元，是2007年的4.75倍。中百、中商、武商、国美、银泰、工贸家电、恒信德龙等知名商家入驻咸宁。物流、信息、中介、会展等新兴服务业竞相发展。三次产业比重由2007年的23.4：42.5：34.1调整为18.6：46.1：35.3。

（二）城乡面貌深刻变化。统筹城乡建设管理，优化城乡发展格局，咸宁的知名度、美誉度和影响度不断提升。

全域咸宁高点规划。坚持以科学规划引领城乡科学发展，统筹推进全域规划的编制实施。市县土地利用总规、城市总规获省政府批复，中心城区规划面积调整到674平方公里，建设面积可达109平方公里。全省首个地级市规划展览馆建成开放。全市总规修编率80%以上，乡镇、行政村规划编制率分别为85.5%、91.5%。咸宁被列为国家“数字城市”地理空间框架建设推广城市。

市区一体共建共管。完善市区一体建管体制机制，策划规划、启动建设“五城二区”，中心城区累计投入155亿元，建成区面积扩大到62.6平方公里，城市功能和产业基础不断完善。潜山国家森林公园、淦河中游综合治理、城区干道刷黑和人行道改造、金桂路、青龙路、南外环、桂乡大道、桂泉互通、武广客运咸宁北站、温泉新客运站等一批重点工程建成投用。实施公交油改气和城区煤改气，开通便民公交线路22条。加大城市创建力度，全面开展立面整治、管网改造、拆违治超、园林绿化工作。建成区绿化率37.6%，人均公园绿地面积10.36平方米。取得了全省“城考”第三、复查第二名的好成绩。国家园林城市、全国旅游标准化示范市创建工作通过国家验收。

城乡一体步伐加快。加快发展县域经济，县域规模以上工业增加值、地方一般预算收入年均分别增长38.4%、25.2%。加强县市城区建设，咸安桂花公园、嘉鱼二乔公园、赤壁城市公园、通城银山广场、崇阳教育新城、通山亲民公园相继建设，嘉鱼县城被评为第三批全国文明村镇。推进新型城镇化和新农村建设，“百镇千村”示范工程与“清洁家园”行动任务全面完成，城乡一体化试点启动实施。咸嘉新城完成规划编制，贺胜“一站一城”规划通过论证，金桂湖低碳发展规划获省批复。8个乡镇（办）进入全省百强乡镇，36个中心村入选全省新农村建设示范村。6个县（市、区）均通高速公路，基本实现了水泥（沥青）路、电话、广播电视、宽带网络村村通，完成了78.96万农村人口安全饮水和76座水库除险加固任务。全市城镇化率由2007年的46.3%提高到49.3%。

（三）民生事业全面进步。牢固树立发展为民的思想，用情用力保民[FS:PAGE]生，尽心尽责促和谐，城乡居民的归属感和自豪感不断增强。

人民生活持续改善。城乡居民人均收入分别由2007年的9991元、3737元提高到14654元和6567元。城镇新增就业15.4万人，农村劳动力转移就业33.8万人，开发公益性岗位8328个。“五险”参保153万人次，是2007年的1.94倍。企业退休人员人均月养老金提高到1123元，是2007年的2.24倍。新农合参合率98.7%，新农保试点参保78.5万人次。被征地农民养老保险稳步推进。

公办基层医疗机构全面实行基本药物制度和药品零差率销售。住房公积金缴存人数达到11万人，市直财政补贴比例提高到工资总额的12%。新建改建配建廉租房6802套，建设经济适用住房2012套，保障困难家庭22180户。实施危房改造23585户，完成因灾倒损民房重建任务13880户。落实城乡低保待遇20.4万人。发放大病医疗救助1.34亿元。完成439个扶贫整村推进、2910户扶贫搬迁任务，解决了13万人的温饱和脱贫问题。通山脱贫奔小康试点扎实推进。库区移民后扶工作得到加强。城乡共建、结对帮扶和“三万”活动成效显著。易肇事肇祸精神病人免费救治、吸毒人员强制收戒工作得到了中央和省委、省政府的充分肯定。未成年人救助保护中心、儿童福利中心和城市社会福利中心加速建设。除王英水库引水工程完成前期、一号桥自来水厂泵站迁移工程已动工外，市政府承诺的其他实事基本完成。

各项事业全面进步。基础教育、职业教育、高等教育和学前教育、特殊教育稳步发展，义务教育免费制度和教师绩效工资制度全面落实，困难学生资助力度持续加大。咸安教师置换培训创造了全国经验，崇阳成为全国留守儿童托管示范县，通山成为全国推行义务教育均衡发展工作先进县。争取省级以上科技项目170个，引进、推广科技成果152项，申请专利1800件。预计高新技术产业增加值22亿元，年均增长21%。咸安、赤壁、通城获评全国科技进步先进县（市、区），赤壁跻身全国科普示范县（市）。建成县级文化信息共享工程6个、乡镇综合文化站67个、村级农家书屋907个、农民体育健身工程335个。《梦寻咸宁》、《白云深处》、《千古风流》、《隽水天城》、《千桥流水桂花香》等地域文化精品纷呈涌现。咸安获批中国嫦娥文化之乡，崇阳获评中国民间文化艺术之乡，汀泗桥镇和羊楼洞村获评中国历史文化名镇（村）。市桂花博览馆、中国楹联长廊建成开放，市博物馆、竹博览馆、传媒大厦、青少年宫、妇女儿童活动中心、奥林匹克体育中心抓紧建设。社会科学、文学艺术、新闻出版、广播影视事业加快发展。外宣工作成效明显。群众性精神文明创建活动蓬勃开展。市供电公司、市实验小学、市国税局和田野集团获评全国文明单位，209个单位评为全省文明单位。重大传染病预防控制工作成效显著。50家乡镇卫生院、712个村卫生室达到标准化建设水平。同济医院托管市中心医院，成为武汉城市圈基本公共服务均等化的示范。计划生育成绩突出，低生育水平保持稳定。社区建设全面加强，71个城市社区全部达到省级示范社区标准。驻咸解放军和武警消防官兵在抗灾救灾、维护稳定、支持发展中作出了突出贡献，民兵预备役工作取得新的进步。民族、宗教、对台、外事、侨务等工作迈出较大步伐。人事、编制、审计、物价、档案、保密等工作不断加强。老龄、青年、妇女、儿童、慈善、残疾人事业全面进步。社会团体在推动经济社会发展中发挥了重要作用。中直、省直驻咸单位在服务地方发展中成绩突出。发改、国土、环保、民政、林业、人行、工商、地税、质监、统计、方志、卫生、人社、计生、老龄、关心下一代、残疾人康复、移动通讯、生物机电成人教育等工作荣获全国性表彰。

民主法治不断加强。自觉坚持党的领导，自觉依照法定权限和程序履行职责，自觉接受人大法律监督、工作监督[FS:PAGE]和政协民主监督，自觉听取各民主党派、工商联、无党派人士和人民群众意见。累计办理人大议案建议288件、政协提案513件，办复率100%，满意率95%以上。不断完善民主决策机制，重大事项集体决策、专家咨询、社会公示和听证、合法性审查等制度得到落实。政务公开全面推进。通山荣获全国政务公开民主管理制度创新奖。基层民主政治建设取得新进展。依法治市工作扎实推进。赤壁荣获全国首批法制县（市）创建活动先进单位。创新社会管理综合治理，信访、维稳和安全工作机制不断完善，应急救援和减灾防灾体系逐步健全，“平安咸宁”建设取得明显成效。

（四）改革开放纵深推进。深化改革，扩大开放，市域发展活力不断增强。

重点改革取得突破。积极推进“两型”社会建设综改试验。“三年行动计划”率先完成。国企改革不断深化，一大批改制企业焕发生机。培育私营企业7152户，发展个体工商户87734户，分别是2007年的2.21倍、1.92倍。农村公益性服务“以钱养事”机制日益完善，农业“小三场”和集体林权制度改革基本完成，水利体制改革不断深化，政策性“三农”保险工作有序推进。规范土地市场管理，土地交易额达120亿元。新增耕地10万亩。通城被评为全国节约集约用地模范试点县。成功引进花旗银行、交通银行、民生银行、吴江农村商业银行、武汉农村商业银行、湖北银行等金融机构，成功组建咸宁市城市建设投资开发有限公司、大通交通投资有限公司、荣盛农业开发有限公司、职业教育投资发展有限公司，组建小额贷款公司6家。农村信用社股份制改革取得突破。咸宁连续九年获评全省金融信用市。市直“两管一治”经验在全省推广。医疗卫生体制改革稳步推进，科技、教育、文化等方面的改革取得重要进展。

开放合作力度加大。累计引资项目1629个，到位资金474.4亿元，其中外商直接投资5.33亿美元，年均增长24.3%。8个省级开发区（工业园区）累计引资到位资金227.4亿元，年均增长30.5%。其中咸宁经济开发区累计引资到位资金68亿元，实现税收5.46亿元，年均分别增长42%、48%。泰国华彬、正大集团，美国捷迈集团，台湾统一、光宝集团，香港华润、中旅集团，中交集团、中投集团等世界500强企业和中广核、碧桂园、南玻、海航、晨鸣、今麦郎等中国500强企业来咸投资。预计外贸出口总额2.3亿美元，是2007年的2.9倍，年均增长30.3%。对外交流合作进一步加强。厅市合作实现全覆盖。援川、援疆和援建建始县工作扎实推进。咸宁被评为全国汶川地震灾后恢复重建先进集体。

政务服务不断改进。顺利完成新一轮政府机构改革。下放、取消、调整行政审批事项465项。行政服务中心规范化建设扎实推进，电子政务、政府网站、政府热线、行风热线、百姓问政、招投标信息发布等政府公共服务平台加快完善。行政执法行为不断规范，综合执法有序推进，市场监管力度加大。政府系统党风廉政建设责任制得到落实。

刚刚过去的2011年，全市上下克服了复杂经济形势和大旱大涝夹击的不利影响，呈现经济较快增长、物价水平趋稳、发展效益提升、民生质量改善的良好态势。预计生产总值增长15.5%，规模以上工业增加值增长26%，全社会固定资产投资增长36.1%，社会消费品零售总额增长19%，出口总额增长36.9%，外商直接投资增长10.1%，地方一般预算收入增长32.5%，城乡居民人均收入分别增长13%和17%，城镇新增就业4.27万人，人口自然增长率控制在6‰以内。“十二五”规划执行实现了良好开局。

各位代表！卓越源自信念，拼搏铸就丰碑。四年来，我们倾注了无限的发展激情。科学确立了鄂南经济强市建设目标，扎实开展了科学发展观学习实践活动，成功举办了三届国际温泉文化旅游节，系统制定了国民经济和社会发展“十二五”规划。四年来，我们亲历了难[FS:PAGE]忘的创业艰辛。奋力夺取了应对国际金融危机、抗击冰雪旱涝灾害的重大胜利，倾力打造了“香城·泉都”的城市品牌。四年来，我们收获了丰硕的发展成果。纵向看，经济总量年度增长突破百亿大关，民生建设和经济发展协调共进；横向比，咸宁成为全省发展较快、变化最大的城市之一，主要经济指标的增幅持续位居全省前列。

各位代表！成绩来之不易，功劳属于人民。咸宁四年来的进步和变化，得益于省委、省政府和市委的坚强领导，得益于历届市（地）委、市政府（行署）打下的坚实基础，得益于市人大、市政协的全力支持，得益于全市人民的团结拼搏。在此，我谨代表市人民政府，向全市广大干部群众，向各位人大代表、政协委员和各民主党派、工商联、无党派人士，向离退休老同志，向驻咸解放军、武警官兵和政法干警，向中直、省直驻咸单位和在咸投资创业的企业家，向所有关心支持咸宁发展的社会各界人士和海内外朋友，致以最崇高的敬意和最衷心的感谢！

各位代表！荣光已成历史，奋斗仍将继续。我们不能忘记发展不够仍是咸宁最大的实际！不能忘记还有42.6万人口没有摆脱贫困！不能忘记还有20.4万城乡居民尚处于低保状态！在前进的道路上还面临不少困难和问题，主要是：综合经济实力和产业竞争力亟待提升，转变经济发展方式任务艰巨；自主创新能力不强，战略性新兴产业尚处于起步阶段；市场体系不完善，服务业发展仍处于初级阶段；城市功能和管理水平有待提高；农村公共服务水平总体较低，农业基础设施薄弱，农民持续增收压力较大；就业、社保、医疗、住房、教育等民生问题仍然突出，社会建设和管理面临新挑战；干部队伍素质、作风与科学发展的要求还有一定差距。我们一定以对人民高度负责的使命感，努力解决存在的突出矛盾和问题，扎实做好政府各项工作，决不辜负全市人民的重托和期望！

二、今后五年工作的总体安排

今后五年，是转型跨越的机遇期、改革开放的攻坚期，也是加快建设鄂南经济强市、全面建设小康社会的关键期。

展望未来五年，使命催生动力。放眼全国，东部发达地区已经或即将率先进入全面小康社会，广大中西部地区都在朝着全面小康社会目标奋力前行。新世纪前十一年，我市在全面建设小康社会的征途上迈出了坚实步伐，鄂南经济强市建设取得了重大进展，但目前我市全面小康程度仍低于全国、全省水平。这就要求我们进一步增强发展的危机感、责任感和能动性，加快赶超步伐，增添跨越实力，在湖北支点建设的大局中立业建功、在区域竞相发展的格局中创优争先。

展望未来五年，机遇大于挑战。从机遇看，全球新的技术革命和产业革命正在孕育，新兴市场国家和发展中国家快速成长；国内经济总体向好，稳增长的主基调和政策框架不会改变；“中部崛起”、“两圈一带”战略和“两型”社会建设综改试验纵深推进，幕阜山区集中连片扶贫开发战略全面实施，长江中游城市群有望成为全国经济增长“第四极”；市域区位、交通、资源、环境等要素优势向经济优势加速转化，“八大试点”效应深度释放，“有中生有”得天独厚，“无中生有”有势可倚！从挑战看，世界经济复苏的不稳定性、不确定性上升，不进则退、慢进亦退的区域竞争态势严峻，在复杂局势中保持经济平稳较快发展的难度更大。这就要求我们审时度势、趋利避害，充分利用重要战略机遇期加快发展，充分利用复杂环境的倒逼机制加快创新，不断开创科学发展的新局面。

今后五年政府工作的指导思想是：以邓小平理论和“三个代表”重要思想为指导，深入贯彻落实科学发展观，按照市第四次党代会的总体部署，以科学发展为主题，以加快转变发展方式为主线，以建设小康、生态、宜居、健康、和谐的幸福美好新咸宁为目标，深化改革，扩大开放，持续[FS:PAGE]实施工业崛起、旅游跨越、城乡统筹、生态持续、人才保障五大战略，全面提升综合实力、创新能力、服务能力和城市竞争力，努力把咸宁建设成为经济繁荣、生态良好、人民幸福、社会和谐的鄂南经济强市。

综合考虑，今后五年经济社会发展的主要预期目标是：地区生产总值年均增长15%；规模以上工业增加值年均增长22%以上；全社会固定资产投资年均增长25%以上；社会消费品零售总额年均增长18%以上，出口总额年均增长15%；外商直接投资年均增长10%以上；地方一般预算收入年均增长20%以上；城乡居民人均收入年均增长13%以上；单位生产总值能耗下降16%，主要污染物排放控制在省定标准以内。人民生活更加殷实，咸宁争得应有地位，为实现全面建设小康社会的奋斗目标打下决定性基础。

实现上述目标，必须牢牢把握三个关键：一是坚持以科学发展观统领经济社会发展全局，在科学发展上下功夫。创新发展理念，优化发展模式，提高发展质量。着力调整经济结构和转变发展方式，着力加强资源节约和环境保护，着力推进改革开放和自主创新，着力促进社会发展和解决民生问题，努力做到速度、质量、效益相协调，投资、消费、出口相协调，人口、资源、环境相协调。二是坚持解放思想、与时俱进、开拓创新，在跨越发展上下功夫。不断开阔视野，勇于探索实践，用发展的办法解决前进中的问题，用市场的手段管理经济，用创新的精神培植新的优势。坚持从咸宁的实际出发，既要因地制宜、苦练内功，更要居安思危、学习先进、跨越发展，基本建成鄂南经济强市。三是坚持以人为本、关注民生、促进公平，在和谐发展上下功夫。统筹城乡、区域、经济社会、人与自然的和谐发展。正确处理改革发展稳定的关系。认真解决人民群众最关心、最直接、最现实的利益问题，真正做到发展为了人民、发展依靠人民、发展成果由人民共享。

今后五年，在全面做好经济社会发展各项工作的同时，重点推动以下五个方面的工作走在全省市州的前列。

（一）加快经济结构调整，在转变发展方式上力争走在全省市州前列。以投资规模的扩大、结构的优化促进产业的调整优化、消费的提档升级，确保投资总规模达5000亿元以上。产业结构调整的重点是提升优化第一产业，做强做大第二产业，大力发展第三产业，形成高端化、高质化、高新化的产业结构。三次产业结构调整为12：48：40。坚持走新型工业化的道路，推进信息化与工业化的深度融合，增强产业的丰厚度与集中度，着力发展一批优势支柱产业、一批大型企业集团和一批知名品牌，打造全省高科技研发转化基地、区域性现代制造业基地。坚持“两增同步”的现代农业发展理念，以农民增收为核心，努力探索产业高效、产品安全、资源节约、环境友好、技术密集、功能多样的农业现代化发展道路。统筹推进特色板块基地建设、农产品加工转化、农民专业合作组织培育、农业基础设施建设、农技推广体系健全等关键环节的工作，建成全省现代农业示范基地。抓住创建全国旅游标准化示范市的机遇，建设一批现代服务业设施，加快完善现代服务业体系，在金融、商贸、现代物流、房地产、中介服务、文化创意等方面体现新的聚集效应，打造全国优秀旅游目的地、全省区域性现代商贸物流基地。

（二）加快城乡一体化建设，在统筹城乡发展上力争走在全省市州前列。坚持以工业化带动农业、城市化改造农村、市民化提升农民，形成城乡统筹、协调发展的新格局。加快建设和完善以港、铁、公、空为重点的现代交通体系，以水、气、电、讯为重点的地下综合管网体系，以中心城区、县市城区、中心镇为重点的城镇体系。增强中心城市首位度，建成区面积达到100平方公里，“五城二区”建设取得实质性进展。咸嘉新城初具雏形。统筹推进县域经济社会发展，重点支持[FS:PAGE]咸安贺胜桥“一站一城”、嘉鱼临港新城、赤壁光谷产业园、通城玉立新城、崇阳“一府一城”、通山通南经济走廊建设；强化城乡规划执行监管，体现规划的先导性、科学性和权威性；以推进产业和人口集聚为重点，以体制机制创新为动力，加快新型城镇化进程，提升新农村建设水平；推动基本公共服务向常住人口扩展，形成南北互动、优势互补、特色鲜明、协调发展的新型城镇体系和现代产业体系。全市城镇化率达60%以上。坚持为了群众、相信群众、依靠群众的理念，共同把咸宁城乡规划好、建设好、管理好。

（三）加快改革创新步伐，在“两型”社会建设综改试验上力争走在全省市州前列。坚持市域经济市场化、经济运行方式股份化、国有集体资产资源资本化、基础设施和竞争性社会事业产业化、政府行为法制化的改革方向，着力构建充满活力、富有效率、更加开放、有利于科学发展的体制机制，拓展“两型”生产生活方式，深化厅市合作内涵，打造武汉城市圈“两型”社会建设综改创新示范基地。大力推进全民创业，稳健发展非公经济。深化政府投融资体制改革。坚持以改革促开放、以开放扩内需，打造中部地区产业转移重要承接基地。全面落实人才发展规划纲要，着力打造人才、科技、产业三者之间的生态链，形成人才引领、科技驱动、产业支撑的发展格局。以全省社会管理创新试点为契机，着力构建民主法治、公平正义、充满活力、和谐稳定的社会局面。

（四）加快生态文明建设，在发展低碳经济上力争走在全省市州前列。牢固树立绿色、低碳、循环发展理念，着力建设以低碳排放为特征的产业体系和消费模式，增强可持续发展能力。严格落实节能减排目标责任制，建立更加有效的监管体系，确保完成节能减排的硬性任务。发展低碳经济，促进绿色增长，加速推进金桂湖低碳示范区建设，建成全省首个低碳经济示范区。积极发展节能环保关键技术装备和产品，加速推进全国新型能源基地建设。抓好农村环境连片整治工作，大力推进碳汇造林和水生态保护与修复。全市森林覆盖率60%，城市建成区绿地率45%。依法强化土地管理，重视土地后备资源开发，提高土地集约化利用水平。

（五）加快公共服务均等化，在提高人民幸福指数上力争走在全省市州前列。加强公共财政建设，推进基本公共服务均等化。千方百计增加居民收入，促进社会财富公平分配。坚持以项目扩大就业、以创业带动就业、以政策扶持就业、以服务保障就业，实现新增就业岗位15万个。逐步建立全覆盖、可选择、多层次、能转接的城乡一体社会保障体系，城镇职工、居民基本医疗保险参保率达96%以上，90%农村适龄居民参加新农保。加快保障性住房建设，改善城乡居民居住条件。加大扶贫开发力度，统筹推进幕阜山区集中连片扶贫开发。坚持优先发展教育，优化教育资源配置，力争教育投入达到地区生产总值的4%。完善医疗卫生服务体系，提升医疗卫生服务水平。加强社会主义核心价值体系建设，推动文化大发展大繁荣。扎实推进文化馆、档案馆、图书馆、科技馆等公共设施建设。大力发展群众体育和竞技体育。市民健康素质达到全国平均水平。

我们坚信，经过五年的不懈奋斗，一个幸福美好的新咸宁一定会崛起在鄂南大地上！

三、2012年政府的主要工作

2012年是实施“十二五”规划的承启之年，是新一届政府的起步之年。特别是党的十八大和省第十次党代会将在年内召开。做好今年的工作，意义重大，影响深远。今年经济社会发展的主要预期目标是：地区生产总值750亿元以上；规模以上工业增加值330亿元以上；全社会固定资产投资700亿元以上；社会消费品零售总额280亿元以上，居民消费价格涨幅控制在4.5%左右；出口总额2.7亿美元；外商直接投资1.9亿美元；地方一般预算收入42亿元以上；城镇居民家[FS:PAGE]庭人均可支配收入16600元；农民人均纯收入7500元；城镇登记失业率控制在4.6%以内；人口自然增长率控制在6.5‰以内；全面完成省定节能减排目标任务。

各位代表！好中求快是我们长期追求的发展状态，稳中求进是中央确定的经济社会发展的总基调。立足咸宁实际，我们既要坚持稳中求进，更要坚持稳中竞进。目前，我市主要经济指标占全省的比重仍低于人口占全省的比重。唯有稳中竞进，才能以快于、重于、优于其他地区的发展绩效开创赶超跨越的新局面，才能更有条件积极回应人民群众的新期待。要认真贯彻落实党的十七大、十七届五中六中全会和中央、省委经济工作会议以及市第四次党代会精神，突出主题，贯穿主线，培基固本，稳中竞进，转型升级增活力，改革开放强动力，统筹发展挖潜力，改善民生聚民力，加快富民强市步伐，以优异的成绩迎接党的十八大胜利召开。

（一）更加注重在转型升级中推动内生增长

着力调整优化产业。大力实施以咸宁经济开发区晋级国家级经济技术开发区、1个以上县域经济开发区晋级省级开发区、6个以上乡镇工业园晋级市级工业园、1家企业上市、新增新口径规模以上企业50家为重点的现代工业培基工程，促进工业集群、集约、集聚发展，确保工业园区主要指标增幅达35%以上。推动电力能源、冶金建材、森工造纸、纺织服装、机电制造、食品饮料等六大支柱产业向产业链两端延伸，力争产值总规模达800亿元以上。积极培育生物医药、现代装备制造、新能源、新材料等新兴产业。加大名牌产品创建力度，新增中国驰名商标2个以上。全力推动和建设咸安森工科技产业基地、嘉鱼重点蔬菜产业基地、赤壁砖茶生产基地、通城油茶产业基地、崇阳鸭业产业基地、通山现代牧业示范基地为重点的现代农业培基工程，调整优化农业结构和区域布局。大力发展粮食生产，力争粮食产量增长5%。加快推进农产品加工“四个一批”工程，实现农产品加工销售收入100亿元以上，新增“三品”认证30个以上。启动建设市茶叶博览馆。推进“山上再造”、秋冬农业开发，实施农村土地整治20万亩，植树造林30万亩。大力实施以百盟欢乐世界、城市商业综合体、天成家居建材城、环三湖连江生态文化旅游区、新华保险养生之都、三国赤壁文化旅游试验区、通城鄂湘赣边贸中心、崇阳天城物流园建设和金融、中介服务创新为重点的现代服务业培基工程，加快服务业集聚区建设。进一步整合旅游资源，深化旅游标准化建设，加大旅游行业创优、旅游名镇名村创建力度，加快温泉游客集散中心、赤壁碧水云谷度假村、河背特大桥湿地公园、太乙养生园、咸安嫦娥谷漂流、通城药姑山旅游、崇阳隽水河温泉、通山大九宫旅游区等项目建设，促进旅游与工业、农业、文化和商贸等产业相融发展，力争接待游客1800万人次、旅游总收入增长35%。壮大金融市场规模，优化金融生态环境，力争存贷款余额分别增长25%以上。加快发展第三方物流、冷链物流等新兴物流业，鼓励发展服务外包。加强普通商品房和保障性安居工程建设，促进房地产业健康发展。支持发展邮政通信业，加速“三网”融合进程。

着力增加有效投资。积极争取政策性投资，不断优化财政投资，努力拓展社会投资，加快推进直接融资，力争固定资产投资增长30%以上。大力实施以武深高速咸宁段、咸黄高速、咸通高速、武咸城际铁路、咸崇旅游公路、金桂湖景观公路、咸潘一级公路为重点的路网建设培基工程，推动嘉鱼和赤壁长江大桥、潘家湾码头、中心城区绕城高速、嘉鱼至赤壁沿江大道规划建设。推动咸宁核电、蒲圻电厂二期、红牛三期、光宝二期、南玻产业园、光谷赤壁产业园、林浆纸一体化、雨润食品、兴民钢圈、红牌电器、新合作鄂南配送中心等项目加快建设，推动今麦郎二期、喜马拉雅光电、三环二期、荣事基业、华宁[FS:PAGE]防腐、菁春生物、洁丽雅二期、脑酷脑饮料、永通发动机等项目提速建设，促进厚福医疗器械、长城碳素二期、楚源生物、惠生药业、金海达建材、华亿通橡胶、盛宇三期等项目达产增效。抢抓机遇，大兴水利，提高农业防灾抗灾能力。充实完善项目库，做好项目申报、争取和实施工作，确保重大项目建设有序接替。

着力扩大消费需求。提升居民消费能力，改善消费预期。加强物价监测预警，完善物价上涨与低收入群众生活补贴联动机制，确保低收入群体基本生活水平稳定。深入实施“万村千乡”、“双百”、“农超对接”、“新网”工程，新建100家村级综合服务社，扩大农村消费规模。继续推进网点进社区、服务进家庭，稳步发展汽车等大宗消费，加快促进城市耐用品消费升级，大力发展物业、家政、培训等领域消费。改造升级运输、商贸、餐饮、住宿等传统消费，稳健发展电子商务、文化娱乐、休闲健身等新兴消费。规范发展会计、律师、公证、监理、资产评估、信息咨询等中介服务。加强综合性物流平台建设，完善城乡流通体系和便民服务设施。优化消费环境，建设绿色市场。

（二）更加注重在改革开放中增强发展活力推进创新型城市建设。强化企业技术创新主体地位，推进高新技术产业、农业产业化基地、科技投融资体系、现代科技园区、科技成果转化基地建设取得新突破，确保高新技术产业增加值增长25%以上，科技进步贡献率55%以上。积极引进研发机构、申报科技专项。与省科技厅合作，开展面向全国科技企业的引资引智活动。加强知识产权保护力度，大力普及科学技术知识。深入推进“两型”社会建设综改试验，放大厅市合作共建效应。建立现代企业制度，完善法人治理结构。健全国有资产监管体制，加快推进政府部门所属企业的脱钩改制。加强财政科学化、精细化管理，完善公共财政体系。进一步创新投融资机制，支持市城市建设投资开发有限公司发行债券6亿元，培育发展面向小微型企业和“三农”的金融机构，组建农村商业银行，发挥保险的融资保障功能。推进要素市场化配置改革。深化农村综合配套改革，创新乡镇运行机制。全面完成文化体制改革任务。深入推进医药卫生体制改革、事业单位分类改革。

推进创业型社会建设。践行“产业第一、企业家老大”的理念，大力培育市场主体。鼓励和引导非公有制经济发展，确保平等使用生产要素、公平参与市场竞争、同等受到法律保护。完善创业扶持政策，健全创业服务体系，推进全民创业，助推企业二次创业，扶持初始创业，引导能人创企业、百姓创家业、干部创事业、外出务工经商人员回乡创实业。营造鼓励脚踏实地、勤劳创业、实业致富的社会氛围。新增个体工商户1万家。

推进开放型经济发展。坚持敢开放、真开放、先开放、全开放、深开放，打造亲商、利商、暖商、敬商、懂商的开放环境。立足本地交通、区位、资源、环境优势和产业基础，加快承接产业转移，加大招商引资和择商引智力度，重点加强对日韩、台港澳、长三角、珠三角、闽三角及环渤海等地区的招商，深化与央企、省属国企的合作对接，着力引进产业龙头项目和产业链配套项目。健全招商项目保障机制，提高项目的落户率、开工率和投产率，力争实际利用内资增长35%以上、外商直接投资增长10%以上。争取海关、商检咸宁分支机构获批。完善出口促进政策，加强出口基地建设，推动出口主体和出口市场多元化。创新“走出去”方式，鼓励有条件的企业对外直接投资，稳步推进境外资源合作开发。

（三）更加注重在统筹发展中强化区域融合统筹推进城乡建设。强化规划引领开发的理念，实现建设区域控规和市域350个中心村试点规划编制全覆盖，提高城乡规划的执行力。强化中心城市的带动功能，在做优“泉都”品牌的同时，加大“香城”品牌的开发建[FS:PAGE]设力度，健全综合交通、园林绿化、城市防洪、停车场所、公共厕所等配套设施，加快推进十六潭公园、香吾山公园、茶花公园、双龙山公园、青龙山公园二期、桂花公园建设和市区污水管网、温泉商场片区、车站路片区、西河桥片区改造等一批重点城建项目，大力加强专业市场、高端市场和特色街区、星级酒店、便民街巷建设，持续提高基础设施承载力、产业聚集配套力和市民生活保障力。加大城市经营力度，规范土地市场，广泛吸引各种资本参与城市建设。进一步创新城市管理机制，加快“城中村”、“城郊村”改造，加快“数字咸宁”建设，全面实施网格化社会管理。立足夯实基础、培育产业、完善功能、提升品位，积极推进新型城镇化。按照每个县市区选择1-2个乡镇、每个乡镇选择1-2个中心村、每个街道办事处选择1-2个社区的原则，大力实施城乡一体化试点建设固本工程。总结推广官桥八组经验，完善村企共建新农村机制。发展壮大村级集体经济，努力化解农村债务。有序引导符合条件的农民工进城落户。

统筹推进区域发展。高昂市区发展龙头，加大“五城二区”建设力度，推动市区一体化发展。支持咸宁经济开发区体制机制创新、产业集聚集群、融资难题破解等工作，推动“三个百亿工程”加快实施，力争市区主要经济指标增幅高于全市平均水平5个百分点。鼓励县市区争资引资、加快发展，在全省县域经济发展年度综合考评中争先进位。抓住长江中游城市群建设和长江经济带新一轮开放开发机遇，大力发展“飞地经济”，加快推进“北三县”沿江经济带建设。抓住幕阜山区集中连片扶贫开发机遇，巩固完善专项扶贫、行业扶贫、社会扶贫“三位一体”扶贫格局，加快推进“南三县”特色经济带建设。深入推进通山脱贫奔小康试点工作。

统筹推进生态文明。严格节能减排工作责任制，实行环境保护“一票否决、诫勉谈话、区域限批、行政问责”四项制度。强化能源资源节约和高效利用，推进节能环保技术开发、示范和推广。大力发展循环经济、低碳经济，大力实施以金桂湖低碳经济示范区、梓山湖生态新城和咸宁经济开发区生态园区建设，陆水河流域修复治理，西凉湖、斧头湖、黄盖湖综合治理，以及生物质发电、垃圾焚烧发电、污水处理等项目为重点的生态环境建设固本工程，支持赤壁华润电力、通山大盘垅工业园建设循环经济示范园。全面完成农村环境连片整治试点任务。创新用地保障机制，加大用地清理规范力度，稳步推进迁村腾地、土地增减挂钩等工作。加大山体、水体和林地、湿地保护力度。率先建成全省首家环保模范城市，积极申报创建国家文明城市、国家卫生城市、国家生态园林城市、国家森林城市、国家环保模范城市。

（四）更加注重在改善民生中促进社会和谐强化保障惠民。全面落实惠民政策，不断改善城乡居民生产生活条件。大力实施以促进城乡就业、社保扩面提标、困难群体救助、保障性住房建设、“万名干部进万村挖万塘”活动、生产安全与食品药品安全、校园安全与校车安全为重点的民生保障固本工程。建立健全职工收入稳定增长机制，完善津补贴政策，提高最低工资标准和最低生活保障水平。实施更加积极的就业政策，加大创业促进就业力度，推动城乡居民就业与本地企业用工的需求对接。扩大社会保险覆盖面，力争“五险”参保率90%以上。统筹做好被征地农民社保工作，落实企业退休人员基本养老金调标、医疗保险提高待遇等工作，确保城镇居民医疗保险和新农合实现全覆盖。推动县（市、区）加大住房公积金财政补贴力度，力争补贴标准达到工资总额的8%以上。完善城乡低保制度，加强社会救助工作。大力推进保障性住房建设。加强“米袋子”和“菜篮子”工程建设，全力保障市场供给。加强城市供水、供气市场监管，完成一号桥自来水厂泵站迁移工作。加强和创[FS:PAGE]新社会管理综合治理，更加注重服务为先、依法管理、源头治理、社会参与。坚持用群众工作统揽信访工作，切实解决群众合法合理诉求。健全打防控一体化的社会治安防控体系。健全应急管理体系，提高应急处突能力。

强化文化惠民。坚持用社会主义核心价值体系引领社会思潮，提高公民思想道德水平和科学文化素质。完善公共文化服务体系，推进文化场馆建设，促进文艺精品的创作与生产。大力实施以市传媒大厦、奥林匹克体育中心、文化艺术中心、潜山古寺恢复、向阳湖名人文化、咸安群众文体活动中心、民间文化建设为重点的文化建设固本工程，丰富城乡居民文化生活。加强文物和非物质文化遗产保护。提升歌舞剧团的服务功能。积极开展文艺调演、送戏送书下乡等活动。加快发展创意设计、现代传媒、动漫游戏、新闻出版、广播影视、广告会展等文化产业，鼓励和引导社会资本参与文化产业，形成一批骨干企业。强化文化市场监管，净化文化市场。广泛开展全民健身运动，加快发展竞技体育。积极发展哲学社会科学。提升对外宣传水平，注重运用新兴媒体提高舆论引导能力。完善新闻发言人制度。

强化事业惠民。优先发展教育，增加教育投入。优化教师队伍建设，改善城乡义务教育教师居住条件，不断提高教师待遇水平。均衡发展义务教育，普及发展高中阶段教育，积极发展学前教育，努力办好特殊教育和继续教育。加强湖北科技学院、咸宁职业技术学院市场导向性办学，推动咸宁职业教育（集团）学校加快建设。大力实施以企业家、行业领军人才、专业技术人才、高技能人才、农村实用人才、急需特殊人才为重点的人才开发建设固本工程，促进人才优化配置。加强基层卫生服务机构建设，加快全科医生培养，抓好重大传染病和各类职业病防治工作，强化医疗服务管理指导。综合治理出生人口性别比偏高问题，提高出生人口素质，稳定低生育水平。加强民兵预备役建设，做好国防动员和双拥共建工作。健全社会养老服务体系，加大老年活动中心投入，发展老年社会福利事业。依法保障未成年人、残疾人权益，为0—6岁残疾人提供抢救性康复救助。为困难农民工提供法律援助。发挥工会、共青团、妇联等人民团体在社会管理服务中的作用。推进老年公寓、妇女儿童活动中心、青少年宫等项目加快建设。加大城乡社区建设力度。加强国家安全和保密工作。充分发挥气象、水文、防震等工作在防灾抗灾减灾救灾中的作用。继续做好民族、宗教、外事、侨务、对台等工作，进一步加强人防、人事、编制、统计、档案、方志等工作。全面完成援疆、援建工作任务。

强化实事惠民。本着普惠、积极、当年的原则，继续为城乡居民办好十件实事。（1）新增城镇就业3.5万人，农村劳动力转移就业3万人，完成各类职业技能培训2.6万人。（2）完成60个贫困村扶贫整村推进年度任务，帮助4.2万贫困人口脱贫。（3）整治10028口当家塘堰；新解决7万农村人口饮水安全问题。（4）切实加强市区城市防洪工作，全面启动实施淦河下游综合整治工程。（5）建设廉租房2204套，发放廉租房租赁补贴4800户，棚户区改造6597户。（6）建设农村中小学教师周转宿舍300套。（7）全面免费开放城乡公共图书馆、文化馆、纪念馆、综合文化站，新建50个农村健身场所、49个农家书屋。（8）完成150个标准化村卫生室新改扩建任务。（9）确保城乡居民社会养老保险实现全覆盖，参保达60万人以上。（10）投入资金2000万元，继续抓好市区背街小巷的综合治理工作，提升市民的生活舒适度。

我们坚信，在激情奋进的2012年，咸宁的发展前景一定会更加美好，人民的福祉安康一定会不断增进！

四、建设为人民谋幸福的政府

面对建设幸福美好新咸宁的光荣使命，全市政府系统的广大工作人员务必心存感恩、心存要务[FS:PAGE]、心存敬畏，全心全意为人民谋幸福。

（一）始终保持执政为民的政治本色

始终牢记政府一切权力来自人民，视群众呼声为第一信号、群众需求为第一选择、群众满意为第一标准，作决策充分尊重民意，办实事充分体现民需，抓改革充分保障民利。始终牢记群众利益无小事，切实解决好群众最盼、最急、最忧、最怨的问题。更加注重关心城乡困难群众的生产生活，确保新增财力更多地用于民生建设。始终牢记人民群众是真正的英雄，坚持问政于民、问计于民、问需于民。完善调查研究、为民办事、访贫问苦、民主评议等制度，拓宽服务领域，改进服务方式，着力打造群众随时看得见、叫得应、用得上的民本政务。

（二）始终保持奋发有为的精神状态

始终坚持和维护党的团结统一，不折不扣地贯彻落实中央、省委和市委的决策部署。始终保持昂扬向上的朝气，把精力集中在想干事上，把本领体现在会干事上，把目标锁定在干成事上，燃烧激情，开创新局。始终保持先行先试的勇气，解放思想、勇于开拓，创造性地推进经济社会发展，没有条件创造条件，没有路子闯出路子。始终保持争创一流的锐气，自觉提高标杆，层层传递压力，在扬长补短中增创优势，在超越自我中寻求突破，力求发展速度能快则快、发展目标能超则超，形成你追我赶、竞相发展的生动局面。

（三）始终保持务实高效的工作作风

坚持干实在事、说实在话、做实在人，开短会、讲短话、发短文、减应酬，把精力更多地用于帮助基层化解难题、推动发展上。坚持雷厉风行抓落实，对定下来的事情立说立行、办就办好。“宁愿政府麻烦，不让百姓埋怨”，完善现场办公、服务承诺、限时办结等制度，全面提升行政服务品质。完善行政服务中心和公共资源交易中心的建设管理，创新行政审批方式，完善并联审批、公开承诺、“一表制”、联合年检等制度，提升行政审批效能。坚持不懈地推进“治庸问责”，以目标责任制为抓手，统筹整合各类检查评比工作，改进政府绩效管理，以发展论英雄，凭实绩定优劣，让实干者无悔，让开拓者无忧。

（四）始终保持依法办事的行政理念

进一步推进依法治市工作，努力提高依法行政的能力和水平，切实解决执法不严、执法不公、多头执法、粗暴执法等群众反应强烈的问题，确保公正执法、文明执法。维护司法公正，支持审判机关和检察机关依法独立行使审判权和检察权。完善集体决策和决策咨询、公示听证、风险评估和责任追究机制，健全规范性文件合法性审查制度，提高行政决策的科学化、民主化、法治化水平。自觉接受人大法律监督、工作监督和政协民主监督，充分听取各民主党派、工商联、无党派人士以及各人民团体的意见和建议，全面推进政务公开，广泛接受新闻舆论和社会公众监督，确保行政权力依法、公开、公正运行。

（五）始终保持清正廉洁的从政操守

切实珍惜人民的信任，始终牢记肩上的责任，为党尽忠，为民尽孝。切实落实党风廉政建设责任制，积极构建政府系统教育、制度、监督并重的惩治和预防腐败体系，努力从源头上、根本上、机制上预防腐败。切实强化行政监察、经济责任审计等监督制约机制，加强对政府投资项目资金使用、工程招标投标、土地出让开发、财政转移支付、政府采购和国有资产转让等方面的监督，坚决纠正损害群众利益的行为，坚决查处违法违纪案件；牢固树立过紧日子的思想，严格控制一般性支出，加强“三公”经费支出管理，着力打造廉洁政府。

各位代表！咸宁在崛起，跨越正当时。让我们更加紧密地团结在以胡锦涛同志为总书记的党中央周围，在省委、省政府和市委的坚强领导，市人大、市政协和社会各界的监督支持下，紧紧依靠全市人民，踏实走好每一步，激情跨越每道关，为共创幸福美好的[FS:PAGE]新咸宁而努力奋斗！
